# Experimental strategies to improve drug-target identification in mass spectrometry-based thermal stability assays

**DOI:** 10.1038/s42004-023-00861-1

**Published:** 2023-04-06

**Authors:** Clifford G. Phaneuf, Konstantin Aizikov, Dmitry Grinfeld, Arne Kreutzmann, Daniel Mourad, Oliver Lange, Daniel Dai, Bailin Zhang, Alexei Belenky, Alexander A. Makarov, Alexander R. Ivanov

**Affiliations:** 1 Barnett Institute of Chemical and Biological Analysis, Department of Chemistry and Chemical Biology, Northeastern University, Boston, MA USA; 2 Sanofi, Disease Profiling and Functional Genomics, Cambridge, MA USA; 3 Thermo Fisher Scientific, Bremen, Germany; 4 Tarmeta Biosciences, Natick, MA USA

**Keywords:** Proteomics, Target identification, Mass spectrometry, Proteomics, Target identification

## Abstract

Mass spectrometry (MS)-based thermal stability assays have recently emerged as one of the most promising solutions for the identification of protein-ligand interactions. Here, we have investigated eight combinations of several recently introduced MS-based advancements, including the Phase-Constrained Spectral Deconvolution Method, Field Asymmetric Ion Mobility Spectrometry, and the implementation of a carrier sample as improved MS-based acquisition approaches for thermal stability assays (iMAATSA). We used intact Jurkat cells treated with a commercially available MEK inhibitor, followed by heat treatment, to prepare a set of unfractionated isobarically-labeled proof-of-concept samples to compare the performance of eight different iMAATSAs. Finally, the best-performing iMAATSA was compared to a conventional approach and evaluated in a fractionation experiment. Improvements of up to 82% and 86% were demonstrated in protein identifications and high-quality melting curves, respectively, over the conventional approach in the proof-of-concept study, while an approximately 12% improvement in melting curve comparisons was achieved in the fractionation experiment.

## Abstract (web summary)

Mass spectrometry-based thermal stability assays (MS-TSA) are a promising approach to characterize protein-ligand interaction, and several strategies have been recently developed to improve their performance. Here, the authors combine three recent strategies to qualitatively and quantitatively improve aspects of MS-TSA.

## Introduction

The assessment of cellular-level target engagement and off-target binding is highly relevant to drug development^1^. A low success rate in clinical trials and the high cost of drug candidate approval resulted in prioritizing target engagement and identification strategies in pharmaceutical companies in recent years^2–4^. One of the most promising contemporary approaches to overcome these challenges is the characterization of drug-protein interactomes using thermally-challenged sample sets in combination with untargeted mass spectrometry. The seminal mass spectrometry-based technique known as thermal proteome profiling (TPP)^5^, followed by MS-based cellular thermal shift assay (CETSA MS)^6^ (collectively, here we refer to both approaches as mass-spectrometry-based thermal stability assays, MS-TSA), are adaptations of the CETSA technique^7^, which exploited the phenomenon of ligand-induced thermal stabilization of proteins by comparison of protein-level melting curves generated from treated and untreated samples. Briefly, this involved heating sample aliquots of whole cells or cell lysates over a temperature range, digestion, labeling with isobaric peptide tags, and data acquisition using multiplexed-sample mass spectrometry, where each protein melting curve is a composite of peptide-spectral matches (PSM).

Several strategies to improve the performance of LC-MS data acquisition in proteomic profiling of challenging samples have been recently demonstrated and appear suitable for enhancing MS-TSA techniques. For example, Grinfeld et al.^8^ introduced the “Phase-constrained Signal Deconvolution Method (ΦSDM), which has been commercialized by Thermo Scientific as TurboTMT© and was shown to significantly improve mass resolution using shorter transient times^9^.

High-Field Asymmetric-waveform Ion Mobility Spectroscopy (FAIMS) has been reported to interface with LC-MS-based proteomics with the effect of improving precursor ion populations at the MS1 level and reducing the co-isolation of co-eluting peptide ions in MS^2^ scans with similar mass-to-charge (mz^−1^)^10–12^.

Furthermore, the implementation of a stable isotope isobarically labeled carrier channel (SIILCC) has been shown to increase the proteome coverage in studies using Single Cell ProtEomics by MS (SCoPE-MS), while follow-up studies using Boosting to Amplify Signal with Isobaric Labeling (BASIL) and SCoPE2 have shown their benefits when implemented in previously reported MS-TSA studies^13–17^.

We hypothesized that a combination of ΦSDM, FAIMS, and SIILCC may result in the acquisition of higher quality MS^2^ scans in MS-TSA experiments. In this study, we developed an MS-TSA model system for the assessment of small molecule-protein interactions using intact cells treated with a commercially available highly specific mitogen-activated protein kinase (MEK) 1–2 inhibitor to prepare a set of unfractionated test samples labeled with tandem mass tags^18^. Our goal was to identify the optimal iMAATSA combination of ΦSDM, FAIMS, and SIILCC to improve the qualitative and quantitative aspects of MS-TSA, which we hoped would translate into an increased number of high-quality melting-curves of distinct proteins. Finally, we compared the performance of the optimally identified iMAATSA combination to the conventional approach using the MEK inhibitor model system in a fractionation experiment.

Circumstances that complicate MS-TSA experiments are not limited to sample preparation protocols but also arise during data acquisition from low-quality fragmentation scans, which often result in Tm variations between replicates^5,7,19–22^. Algorithms controlling the “roll-up” process, where PSM quantitative values are typically filtered and either averaged or summed up into peptides’ quantitative values and ultimately for protein groups, are largely undisclosed owing to their proprietary nature as commercialized products, which may also contribute to suboptimal data quality used in the process of building protein-level melting curves. Considering this anticipated issue, we investigated basic PSM-level filtering of our identified MEK2 target to determine if this approach could improve the agreement of T_m_ between replicates.

Here in the proof-of-concept (POC) experiment we demonstrated improvements in protein identifications and high-quality melting curve comparisons of up to 82% and 86% respectively compared to the conventional approach. In the separate fractionation experiment we have shown a 12% improvement in protein melting curve comparisons.

## Results

### Experimental workflow

**Fig. 1:**
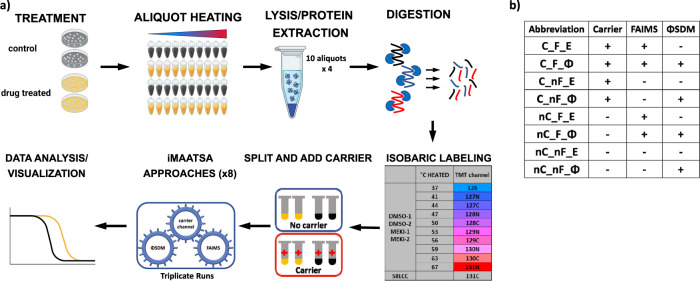
Experimental workflow overview. **a** Jurkat cell cultures were treated in duplicate with either DMSO or the commercially available MEK inhibitor RO4987655, and aliquots of suspended cells were heated to 10 specific temperatures. Detergent was added, and cells were lysed by freeze-thaw and digested overnight with a trypsin-lysC mixture. Aliquots were labeled with TMT10-plex reagents, pooled, and split into two halves, where one half received an addition of 50 µg of SDS-solubilized Jurkat cell lysate as a stable isotope isobarically labeled carrier channel (SIILCC). Samples were analyzed using eight iMAATSA approaches. **b** Abbreviations used for the combinations of technologies for eight iMAATSA that were evaluated.

To establish a model cellular system for the assessment of drug-protein interactions, we used intact Jurkat cells treated with and without a highly specific MEK1/2 inhibitor, which expectedly resulted in changes in the melting temperatures of the target proteins. Compound selectivity was an important consideration in the design of our MS-TSA model system, as a highly promiscuous inhibitor could lead to questionable ΔT_m_ shifts and unreliable conclusions. The use of suspension cells in our MEK model system was beneficial, as cultures were easily expanded with minimal effort and expense, while the MEK1/2 cytosolic kinases were reliably detected without fractionation. Typically, fractionation is required for high proteomic coverage, leading to low experimental throughput; however, the objective of the present work was to assess several experimental strategies for improving MS-TSA performance using both non-fractionated and fractionated samples. The initial POC experiments were conducted without fractionation. Four cell suspensions (two with and two without the inhibitor) were divided into aliquots and heated across a temperature range to exploit the ligand-induced thermal stabilization of targets (Fig. 1a). A non-denaturing detergent was added to each aliquot to aid in cell rupture and membrane protein extraction, followed by cell lysis using several freeze-thaw cycles^23^. Centrifugation was used to remove insoluble debris and isolate the soluble fraction. Acetone precipitation to remove detergent and salts was carried out on identical volumes from each heat-treated aliquot of all samples, followed by digestion. Each digest corresponding to a different heat treatment was then labeled with a unique isobaric tag and then pooled back into their constituent four samples to facilitate multiplexing. The pooled samples were then equally split by volume, and half received a SIILCC. The iMAATSAs with or without a SIILCC are referred to as “C” or “nC,” respectively (Fig. 1b). The SIILCC was prepared by extracting a Jurkat cell pellet containing the same number of cells as each temperature point aliquot with 100 µL 1% SDS. The ability of SDS to solubilize most proteins was the rationale for its use, as we aimed to maximize proteome coverage. In total, eight approaches, including seven iMAATSA approaches and a conventional method that was used as a control (i.e., nC_nF_E), involved various combinations of conditions with and without the use of a carrier channel, ΦSDM, and FAIMS. The iMAATSAs applying or not applying ΦSDM are indicated by “Φ” or “E,” respectively, while applying or not applying FAIMS is designated with “F” or “nF,” respectively (Fig. 1b).

### Accurate melting curves can be produced using a resolution setting of 15 K with ΦSDM

**Fig. 2:**
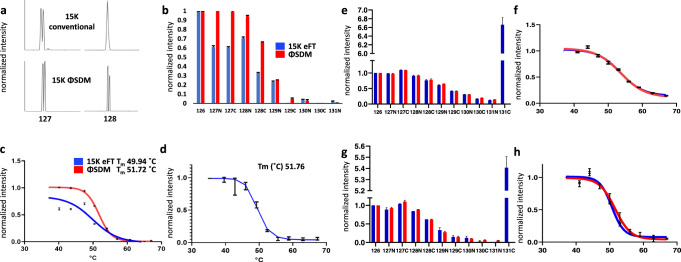
Feasibility of using ΦSMD and stable isotope isobarically labeled cell culture (SIILCC). **a** An example of reporter ion isotopologues ± ΦSDM. Insufficient resolving power results in reporter ion coalescence (top, for illustrative purposes, data was collected in profile mode) using the conventional eFT mode of MS data acquisition, while the ΦSMD-based approach allowed for resolving closely spaced reporter ions. **b** Proportional heat response was not observed in MEK2 reporter ion intensities acquired with conventional eFT MS data acquisition at 15 K resolution setting compared to MEK2 reporter ion intensities acquired at 15 K resolution setting using ΦSDM. **c** MEK2 melting curves acquired with and without ΦSDM show differences in T_m_ and melting curve profiles. **d** MEK2 melting curve from the “Meltome” database as a reference melting curve. **e** Median reporter ion intensities acquired with and without SIILCC demonstrating the negligible effect of the SIILCC carrier channel on the performance of TSA measurements. **f** Median fitted protein level melting curves with and without SIILCC showing high similarity. **g** MEK2 reporter ion intensities acquired with and without SIILCC. **h** MEK2 fitted protein level melting curves with and without SIILCC demonstrating high similarity in curve profiles and T_m_ values. The error bars represent the standard deviation (*n* = 2 for **e** and **g**, and *n* = 3 for **b**, **c**, **f**, and **h**).

Faster data dependent acquisition (DDA) cycle times resulting in increased throughput in producing MS^2^ scans with sufficient ion statistics are preferred in MS-TSA experiments as this will lead to an increased number of protein melting curve comparisons. The 30 K resolution setting at mz^−1^ 200 for acquiring MS^2^ spectra by eFT data acquisition on Orbitrap instruments, which is commonly used for acquisition of MS^2^ spectra in enhanced Fourier transform (eFT) data acquisition, leads to a transient duration of ~64 ms, while utilizing a lower resolution setting of 15 K (at mz^−1^ 200) results in a shorter transient duration of ~32 ms and higher MS^2^ scan rates^8^. We hypothesized that the recently developed ΦSDM approach implemented at a 15 K MS^2^ resolution setting might increase the depth of MS-TSA profiling compared to the conventionally used 30 K resolution setting required for eFT-based data acquisition. An experiment using a resolution setting of 15 K, either with or without ΦSDM, was conducted to determine the feasibility of a low-resolution setting of 15 K for iMAATSA. We confirmed previous findings^9^ that insufficient resolving power for ^15^N and ^13^C TMT 10-plex reporter ions resulted in a failure to separate reporter ion isotopologues at a resolution setting of 15 K when using eFT, as exemplified in (Fig. 2a). An *actual* resolving power exceeding 20 K for the TMT reporter ion mz^−1^ range is required for resolving the ^13^C and ^15^N TMT channels. Therefore, the 32 ms transients with eFT appear insufficient for resolving such TMT signals. However, the ΦSDM enhancement of resolution makes these transients usable for reliable TMT-based quantitation. MEK2 reporter ion intensity profiles were compared using 15 K ΦSDM and 15 K eFT, and in both cases, despite showing high reproducibility in repeat injections, a clear heat response trend was only observed in the 15 K ΦSDM profile (Fig. 2b). The incongruous melting profile of MEK2 at 15 K eFT is likely a result of partial ion coalescence. The MEK2 reporter ion intensities were then fitted to Boltzmann sigmoidal nonlinear models with resulting T_m_ values of 49.94 and 51.72 °C for eFT and ΦSDM, respectively (Fig. 2c). A difference of 0.04 °C was found between the T_m_ of the ΦSDM fitted MEK curve, and the “Meltome” resource^24^ (Fig. 2d), while the difference between the T_m_ values determined by eFT and reported in “Meltome” was 1.82 °C. The T_m_ difference for MEK2 between the two approaches here was relatively small; however, the precarious nature of ion coalescence and the melting profiles observed in the eFT approach could conceivably yield much greater differences in other cases.

### The use of an isobaric carrier channel does not alter melting-curve profiles or T_m_ determination

Next, we evaluated the feasibility of including a labeled SDS-solubilized whole-cell lysate isobaric carrier sample in iMAATSA experiments to eventually be used to increase the amount of distinct protein melt-curve comparisons. The greater the proteome coverage, the more likely unknown targets or off-targets will be identified in MS-TSA experiments. We anticipated the use of a SIILCC would not modulate the accuracy of peptide quantitation for generating melting curve profiles or compromise the ability to detect thermal shifts. MS-TSA methods rely on the separation of soluble from heat-denatured and precipitated proteins, precluding the use of strong denaturing detergents such as SDS, which is commonly used to keep proteins in solution during the heated reduction step before digestion, and improve membrane protein extractions. However, SDS can be used to solubilize a carrier channel proteome, as this channel would not be used in melt-curve processing but instead to increase the recovery of lower abundance proteins or proteins with low aqueous solubility as seen in SCoPE-MS and BASIL protocols. It has been recently reported^25^ that the use of a lower level of carrier proteome is beneficial to minimizing CV (%) in quantitative analyses. We chose to use a carrier proteome in the 131C-TMT channel at 4-times the amount compared to the amount of total protein in the 37 °C heated sample to prevent TMT isotope impurities from adversely affecting the shape of melting curves; however, further studies are needed to identify optimal amounts or SIILCC in MS-TSA settings. Median reporter ion intensities from individual heat-treated channels for all detected proteins were used to fit a “median” melting curve in order to assess global changes at the whole proteome-level as a result of carrier channel spike-in. MEK2 was used to monitor changes within the individual target protein level in a similar way. Median normalized reporter ion intensities for the experiment with the carrier channel, C_nF_E, and the control nC_nF_E matched well (Fig. 2e), and the median intensity of the carrier channel was approximately 6.5-fold higher than the intensity of the 126 channels, which corresponded to the lowest heat treatment (i.e., 37 °C). After fitting the median normalized reporter ion intensities for all detected proteins to a Boltzmann sigmoidal model, the mean melting temperature difference was found to be <0.1 °C for both C_nF_E and control, nC_nF_E (Fig. 2f). Normalized intensities of MEK2 reporter ions for the C_nF_E and control conditions were also shown to be highly similar (Fig. 2g), resulting in an insignificant mean melting temperature difference of ~1 °C (Fig. 2h), which is typical of T_m_ differences between replicates of proteins observed in our data set and previously reported work^5^.

### The use of iMAATSA approaches improved depth of quantitative proteomic profiling

**Fig. 3:**
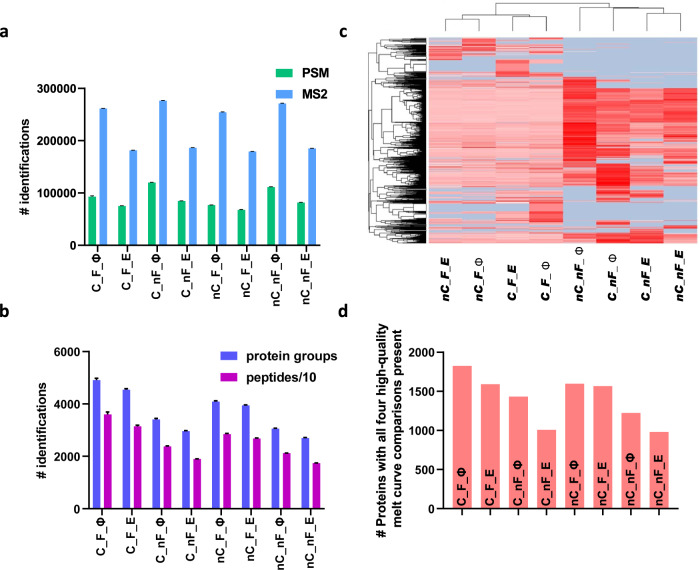
iMAATSA performance evaluations. **a** Comparison of MS^2^ and PSM identifications for the evaluated approaches. **b** Protein group and peptide group identifications in the assessed iMAATSA techniques. SIILCC, FAIMS, and ΦSDM increased both protein and peptide identifications. **c** Heatmap and clustering (using Euclidean distance and complete linkage method) showing protein abundances from 126 reporter ion channel (37 ˚C) in all iMAATSA approaches. Grey represents missing values. The iMAATSA without FAIMS display more missing values but, overall, a higher signal intensity compared to iMAATSA implementing FAIMS. **d** High quality melting curve comparisons for the iMAATSA approaches. The use of ΦSDM, FAIMS, and SIILCC produced the highest number of melting-curve comparisons and identified MEK1 and MEK2 as having a significantly shifted T_m_. The error bars represent the standard deviation (*n* = 4) for panels a and b.

In order to understand the impact of the eight evaluated iMAATSA on the quantity of protein-level melt-curves, we looked at protein, peptide, and PSM identifications, in addition to MS^2^ scans (collectively referred to as “proteomic identifications”). The four iMAATSA implementing the ΦSDM algorithm consistently showed a significant improvement in MS^2^ scans compared to control (Fig. 3a). Most PSMs were identified using C_nF_Φ and nC_nF_Φ (119,922 ± 464 and 111,295 ± 530), while the least PSM identifications came from C_F_E and nC_F_E (75,055 ± 409 and 67,749 ± 715). The highest number of peptides were identified from C_F_Φ with 35,989 ± 826, and nC_F_Φ which produced 28,475 ± 223, while the least number of peptides were found with C_nF_E and nC_nF_E (control) with 18,974 ± 56 and 17,391 ± 62, respectively (Fig. 3b). C_F_Φ and nC_F_Φ identified the most proteins with 4,908 ± 66 and 4,085 ± 31, respectively, while the least protein identifications were found using C_nF_E and nC_nF_E having 2,962 ± 16 and 2,699 ± 16, respectively. The heatmap (Fig. 3c) also supports our findings where C_F_Φ had the least missing values (light green) for 126 reporter ion intensities. However, iMAATSA implementing FAIMS also produced lower signal intensities, which was previously reported in other FAIMS-based applications^26^. While FAIMS removes most singly charged species, allowing the C-trap to fill with more peptides of interest and resulting in increased protein identifications, the increased ion flight path likely contributes to an attenuated signal.

To assess the individual effect of the evaluated technologies, percent improvement compared to their corresponding controls (eFT, no FAIMS, and no carrier channel) was calculated for proteomic identifications. For example, to evaluate the effect of using ΦSDM on protein identifications, the difference between C_F_Φ and C_F_E protein identifications was divided by C_F_E protein identifications and multiplied by 100 to calculate percent improvement, which allowed us to theoretically isolate and compare one technology across four conditions (Fig. S3). Consistently, iMAATSA implementing ΦSDM achieved 42–48% more MS^2^ scans compared to the settings using eFT. PSM identifications improved by 13–42%, and peptide groups by 6–25% using ΦSDM, with the largest gains observed without FAIMS. The highest gains in protein identifications from ΦSDM were seen in the C_nF and nC_nF comparisons with 15% and 13%, respectively. The FAIMS technology demonstrated 34–53% and 34–66% improvements in protein and peptide group identifications but seemed to have a negative effect on PSM and MS^2^ scans, which was previously reported^11^. The use of a carrier channel demonstrated a 10–20% improvement for proteins, 9–26% for peptides, 4–21% for PSM, and 1–3% for MS^2^ scans. Finally, we constructed a heat map showing proteomic identifications for carrier and no carrier separately. Each proteomic identification was compared horizontally across each row. The carrier and no carrier iMAATSA having the most protein identifications implemented both ΦSDM and FAIMS (Fig. S3).

### The use of iMAATSA allowed for significantly more comparisons of high-quality protein melt-curves

**Fig. 4:**
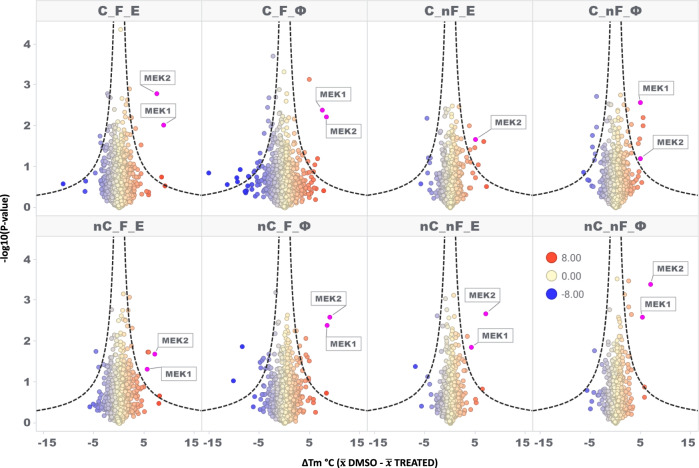
High-quality melting-curves for all eight evaluated iMAATSA identified MEK targets. Volcano plots for each iMAATSA with significance cut-offs (dotted lines). MEK1 and MEK2 demonstrate significant stabilization after treatment with MEK inhibitor RO4987655. The data points represent the results of duplicate experiments.

A successful MS-TSA experiment in the drug discovery setting leads investigators to uncover protein-drug interactions responsible for therapeutic and undesirable side effects (if any) typically by achieving the highest number of protein melt-curve comparisons. We assessed the overall quantity of high-quality protein melting curves as previously described^19^, using our POC unfractionated model system. Briefly, for a protein melt-curve to be classified as high-quality, an R^2^ “goodness-of-fit” statistic value needed to be ≥ 0.8, the slope of the inflection point ≤ -0.06, a lower plateau of ≤ 0.3, and the treated and control melting temperatures be present for both biological replicates. The C_F_Φ iMAATSA, which implements all three evaluated technologies, produced the most high-quality protein melting curve comparisons (1825), while C_nF_E produced the lowest amount in the carrier channel group (1007) (Fig. 3d). A similar trend was observed for iMAATSA without the carrier channel as nC_F_Φ produced the most high-quality protein melting curves (1598), and nC_nF_E produced the lowest amount (981) (Fig. 3d). In order to assess the individual impact of ΦSDM and FAIMS on the quantity of high-quality protein melting curves we calculated percent improvement (as described above) while not assessing the effect of a SILCC because of differences in overall loading amounts. The most significant improvement from ΦSDM came from the C_nF comparison, showing a 42% improvement (Fig. S4a). The most significant theoretical improvements from FAIMS were observed in the C_E and nC_E comparisons showing 58% and 60% improvements, respectively (Fig. S4b). Additionally, we showed that all eight iMAATSA were able to detect such interactions using our MEK model system. MEK1 and MEK2 were observed as significantly shifted proteins, as evidenced in the volcano plots in Fig. 4a, except for C_nF_E, where no unique peptides were identified for MEK1.

### Increased proteomic identifications and melting-curve comparisons were also observed in a fractionation experiment

**Fig. 5:**
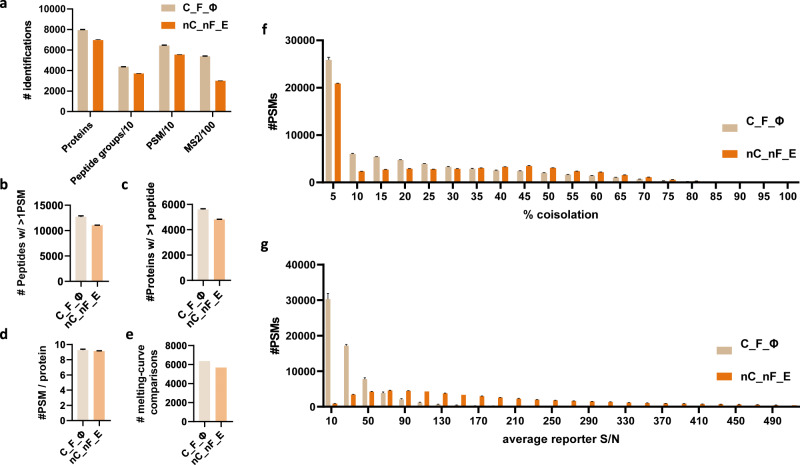
Improvements in the performance of C_F_Φ compared to nC_nF_E shown in the fractionation experiment. **a** Proteomic identifications (proteins, peptide groups10^-1^, PSM10^-1^, and MS^2^ scans100^-1^) from C_F_Φ and nC_nF_E. **b** Number of peptides with >1 PSM, **c** number of proteins with >1 peptides, **d** number of PSMs per protein, and **e** number of melting-curve comparisons for C_F_Φ and nC_nF_E. **f** Histogram (binwidth = 10) of PSM % coisolation for C_F_Φ and nC_nF_E**. g** Histogram (binwidth = 20) of average reporter ion signal to noise (SN) for C_F_Φ and nC_nF_E. The error bars represent the standard deviation (*n* = 3) for **a**–**d**, and **f**, **g**.

The use of unfractionated samples allowed us to conveniently identify the optimal iMAATSA combination; however, a typical MS-TSA relies on fractionation for a comprehensive and deep investigation of detectable protein-ligand binding interactions. We validated our optimally performing iMAATSA (C_F_Φ) and compared it to the control (nC_nF_E) to determine if the benefits would be translated to a fractionation experiment setting using the same MEK inhibitor model system. As shown in Fig. 5a, an improvement was demonstrated in all proteomic identifications using C_F_Φ. Proteins increased from 6,967 ± 46 (nC_nF_E) to 7940 ± 50 (C_F_Φ), peptides increased from 37,062 ± 102 (nC_nF_E) to 43,630 ± 85 (C_F_Φ), PSMs increased from 55,512 ± 96 (nC_nF_E) to 64,420 ± 235 (C_F_Φ), and MS^2^ scans increased from 298,993 ± 853 (nC_nF_eFT) to 537,954 ± 2,728 (C_F_Φ). A > 15% improvement in peptides identified with >1 PSM (Fig. 5b) was observed with 11,019 ± 39 (nC_nF_E), and 12,714 ± 1,565 (C_F_ Φ). Proteins with >1 peptide (Fig. 5c) increased from 4,799 ± 20 (nC_nF_E) to 5198 ± 39 (C_F_ Φ), while PSM/protein (Fig. 5d) showed a > 2% improvement (9.1 ± 0.03 for nC_nF_E, and 9.3 ± 0.09 for C_F_ Φ). The number of melting curve comparisons was assessed using the criteria of proteins having a T_m_ > 37 °C and a calculated melting temperature present for two out of the three replicates in order to calculate a p-value. Using these criteria, an almost 12% increase was achieved in melting-curve comparisons (6364 for C_F_ Φ and 5701 for nC_nF_E, Fig. 5e). Increased identifications of PSMs with lower % precursor ion coisolation values were obtained using the C_F_ Φ iMAATSA. At 35% coisolation, nC_nF_E, and C_F_ Φ had similar % coisolation values. However, at higher % coisolation, we observed more PSMs identified with nC_nF_E, while below 35%, more PSMs were identified with less % coisolation, which we believe is attributable to the use of FAIMS (Fig. 5f). The median average reporter ion signal-to-noise level (SN^−1^) of PSMs for C_F_ Φ was 21.4 ± 1 and 152.4 ± 0.9 for nC_nF_E (Fig. 5g). The higher SN^−1^ of nC_nF_E was likely a result of longer maximum ion injection times.

**Fig. 6:**
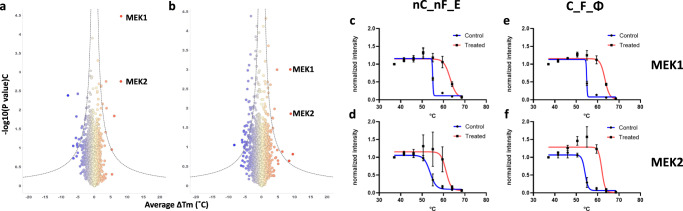
MEK targets identified as having significantly shifted T_m_ in a fractionation experiment. Volcano plots for **a** C_F_Φ and **b** nC_nF_E indicate MEK1 and MEK2 were the most significantly stabilized protein by each iMAATSA. Melting-curves with error bars (*n* = 3) for nC_nF_E of **c** MEK1 and **d** MEK2. Melting-curves for C_F_Φ of **e** MEK1 and **f** MEK2. The data points represent the results of replicate experiments (*n* = 3) for **a**, **b**. The error bars represent the standard deviation (*n* = 3) for **c**–**f**.

MEK1 and MEK2 were identified as having significant a ΔT_m_ in both nC_nF_E and C_F_ Φ (Fig. 6a, b). A ΔT_m_ of 8.12 °C for MEK1, and 7.48 for MEK2 was determined using nC_nF_E (Fig. 6c, d), while a ΔT_m_ of 8.47 and 7.86 °C for MEK1 and MEK2 respectively was determined using C_F_Φ (Fig. 6d–f).

### Signal intensity and average reporter ion SN^−1^ at the lowest temperature treatment thresholds can decrease variability for an identified target’s melting temperature

**Fig. 7:**
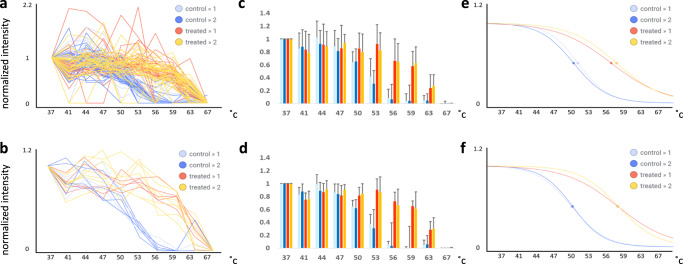
Assessment of signal intensity and average reporter ion SN^-1^ at the lowest temperature treatment thresholds can decrease variability for an identified target’s inflection point. Top panels include all PSMs, while bottom panels have no shared peptides, an average repoter ion SN^-1^ of ≥ 10 and a 126-reporter ion abundance of > 70,000. **a** Overlay of all PSMs corresponding to MEK2. **b** Trendlines plotted for MEK2-derived PSMs excluding PSMs corresponding to (i) shared peptides, (ii) average reporter ion SN^-1^ ratio < 10, and (iii) abundance of TMT 126 reporter ion < 70,000. Mean normalized PSM values for each temperature point for all PSMs **c**, and after filtering **d**. The mean normalized PSM values for MEK2 were fitted to a logistic regression model with inflection points shown as circles without **e** and with filtering **f**.

MS-TSA experiments often include duplicate treated and control samples where replicate T_m_ are rarely in exact agreement. Protein-level data in commonly used proteomic data analysis software platforms (e.g., Proteome Discoverer) is a composite of identified PSMs. Therefore, we hypothesized that low-quality MS^2^ spectra and the resulting PSMs could potentially be a source of T_m_ variability. Here, we looked at the target protein MEK2 and overlaid all PSMs for both vehicle and treated samples for both biological duplicates, as shown in Fig. 7a. While most of the normalized intensity levels of overlaid PSM profiles for drug-treated (red and orange), and control (dark and light blue) show a clear separation in the 53–63 °C temperature range, many PSM outliers are also apparent. In contrast, PSM profiles using only unique peptides, PSMs with a minimum average reporter ion signal to noise (SN^−1^) of 10, and a minimum intensity level for the TMT 126 reporter ion channel (37 °C) of ≥ 70,000, were overlaid (Fig. 7b) and produced an unambiguous separation over the same 53–63 °C temperature range. The mean normalized intensity for each temperature point was calculated for the duplicate control and treated samples, and the standard deviations shown in the error bars are generally reduced, with the exception where mean values were close to zero (Fig. 7c, d). The mean normalized PSM intensities were then used to fit a protein-level melting curve for MEK2 (Fig. 7e, f), which resulted in closer replicate inflection points (used as T_m_ proxy). It should be noted that MEK1 and MEK2 are highly homologous^27^ and share many identified peptides. The effect of filtering of non-unique or shared peptides might be less pronounced in the MEK1/2 case, as opposed to other targets/off-targets with lower homology.

## Discussion

A critical challenge in the drug development pipeline has historically been low success rates in phase II, where the quality of drug target selection is tested broadly in a real-world setting and has proved to be a key bottleneck^2–4^. The MS-TSA approaches of CETSA MS and TPP have successfully demonstrated a capacity to aid drug discovery efforts;^20,28^ however, increased qualitative and quantitative proteomic coverage^23,29^ remain to be improved. Here we have shown that the combination of ΦSDM, FAIMS, and SIILCC inclusion in iMAATSA had an additive effect on performance, facilitating an increase in the numbers of protein identifications and high-quality protein melting curves compared to the control in both non-fractionated (higher throughput but lower profiling depth) and fractionated (lower throughput but increased profiling depth) types of the assessed workflows.

We have demonstrated that implementation of the ΦSDM FT algorithm allows for accurate quantitation using MS data acquisition at a lower resolution setting of “15 K”, which results in an increased MS^2^ duty cycle that is in good agreement with previous work^26^. The ΦSDM FT algorithm by itself increased unique peptide and protein group identifications by 22% and 13%, respectively, compared to the control in the POC experiment. MS-TSA is often not sample-limited, and we expect a correlative benefit from ΦSDM when using increased sample amounts and more fractions. We have successfully included an SDS-solubilized cell lysate SIILCC in a MS-TSA melting-curve experiment for the first time to the best of our knowledge. The T_m_ of both MEK2 and the median protein melting curves were not significantly altered because of the addition of a SIILCC. We expect this approach will assist in the identification of lower abundance peptides to allow for protein melting curve comparisons to be made for lower abundance proteins. We showed the iMAATSA, which implemented all three investigated technologies identified the highest number of protein groups and unique peptides with 4,908 and 35,989, respectively, compared to 2699 and 17,391 protein groups and unique peptides, respectively, compared to the control (i.e., nearly doubled the numbers of identifications). Similarly, the iMAATSA implementing all three evaluated approaches also resulted in substantially superior performance in terms of high-quality protein melting curve comparisons, with 1825 compared to 981 for the control experiment. Moreover, we have highlighted the MS-TSA limitation that protein melting curves can be adversely affected by low-quality/outlier PSMs. Finally, we have shown several first steps to improve the melting curve reproducibility by PSM filtering.

In conclusion, we have combined and implemented three recently developed technologies and shown they have a complementary effect on MS-TSA data acquisition. We recommend using the described approaches based on the sample availability and the desired throughput and profiling depth. For example, the C_F_Φ iMAATSA can be used to increase protein melting curve comparisons in drug discovery applications where sample amounts are either adequate for fractionation or only sufficient for one-dimensional pilot investigations of non-fractionated samples to achieve the highest number of protein melting curve comparisons.

## Methods

### Materials and reagents

50 cm × 75 µm I.D. analytical column ES803, trap column PN 164564, UltiMate 3000 RSLC nano-liquid chromatography system (nLC), Pierce™ Peptide Desalting Spin Columns, Tandem Mass Tag (TMT™) 10-plex reagent kits, triethylammonium bicarbonate (TEAB), tris(2-carboxyethyl)phosphine (TCEP), tris(hydroxymethyl)aminomethane (Tris), sodium dodecyl sulfate (SDS), Titer Plate Shaker (model #4625), HALT protease and phosphatase inhibitor cocktail, Veriti thermocycler, NP-40 Surfact-AmpsTM detergent, MicroAmp^TM^ optical adhesive film, FBS, formic acid (FA), Penicillin-Streptomycin (10,000 U mL^−1^), T225 flasks, 1× PBS, and all solvents at Optima LC-MS grade were purchased from Thermo Fisher Scientific (Waltham, MA) Jurkat E6.1 leukemic T-cell lymphoblasts were purchased from the American Type Culture Collection (ATCC, Manassas, VA). Acetone, 2-chloroacetamide (CAA), RPMI-1640, and dimethylsulfoxide (DMSO) were purchased from Sigma-Aldrich (St. Louis, MO). 96-well semi-skirted Hard shell PCR plates were purchased from Bio-Rad (Hercules, California). Benzonase nuclease was purchased from MilliporeSigma (Burlington, MA). Thermomixer® C was purchased from Eppendorf AG (Hamburg, Germany). Trypsin/Lys-C mix was purchased from Promega (Madison, WI), Cytiva (Emeryville, CA, USA) Sera-Mag SpeedBeads™ Carboxyl Magnetic Beads (hydrophobic and hydrophilic) were purchased from Fisher Scientific.

### Jurkat cell culture

Jurkat E6.1 leukemic T-cell lymphoblasts were grown in T225 flasks containing RPMI-1640 supplemented with 10% FBS and 100 U mL^−1^ Penicillin-Streptomycin in a 37 °C incubator with 5% CO_2_. Cells were cultured and maintained at a density of 1 × 10^6^ cells mL, and fresh media was exchanged the day before compound treatment. Prior to experiments, cells were washed with 1× PBS twice and aliquoted to 96-well PCR plates. Either DMSO or a commercially available MEK inhibitor RO4987655 (MedDChemExpress, 1 Deer Park Dr, Suite Q, Monmouth Junction, NJ 08852, USA) was added, which resulted in a final concentration of 10 µM compound and 1% DMSO. Plates were sealed with MicroAmp^TM^ optical adhesive film, and a 1-hour incubation was performed at 37 °C. Immediately following, the plates were heat-challenged at ten temperature points for the POC experiment, and eight temperature points for the fractionation experiment (37, 41, 44, 47, 50, 53, 56, 59, 63, and 67 °C) for 3 min in a thermocycler and allowed to equilibrate to room temperature on the benchtop for 3 min. To each well, NP-40 Surfact-Amps^TM^ detergent was added for a final concentration of 0.4% and mixed by pipetting. Samples were snap-frozen in liquid nitrogen for 2 min and thawed in a room temperature water bath at room temperature for 2 min for a total of four cycles and stored at −80 °C. A mixture of nuclease, protease, and phosphatase inhibitors was added to each sample for a final dilution concentration of 25 U mL^−1^ and 1×, respectively, and incubated at 4 °C for 20 min on a titer plate shaker at half maximum speed. The lysates were transferred to 8-well PCR strips and centrifuged for 40 min at 11,700 × *g* to remove the precipitate. Subsequently, 80 µL of supernatant from each well was transferred to a new set of 8-well PCR strips and stored on ice. Total protein concentration was estimated using BCA assay according to the manufacturer’s protocol, and samples were stored at −80 °C before digestion.

### Digestion and sample processing

Equivalent volumes from each heat-challenged temperature point that corresponded to 25 µg from the 37 °C aliquot of each replicate were aliquoted into new tubes. SDS and Tris were added for final concentrations of 1% and 100 mM at pH 8, respectively, and each sample was brought to a volume of 106.25 µL with deionized water. Reduction and alkylation were performed in a one-step process using a final concentration of 20 mM TCEP and 40 mM CAA in each sample, followed by heating at 57 °C for 1 h in a thermomixer.

Protein precipitation for the POC experiment was accomplished by the addition of six volumes of ice-cold acetone to each sample, followed by overnight incubation at −20 °C. The following day samples were pelleted by centrifugation at 12,000 × *g* for 15 min at – 4 °C, and the supernatant was removed. Samples were washed with 800 µL of acetone and again centrifuged as previously described. The samples were then dried briefly with the cap open for ≤ 5 minutes, followed by the addition of 50 µL of 100 mM TEAB (pH 8.5) and trypsin/LysC at a ratio of protein to the enzyme of 50:1 and incubated in the Thermomixer® C at 37 °C 500 rpm for four hours. A second aliquot of trypsin/LysC was added for a final ratio of protein to enzyme of 25:1, followed by overnight incubation.

Samples for the fractionation experiment were prepared similarly in 96 deep-well plates (sample plates), except detergent removal was accomplished using an SP3 magnetic bead approach (instead of acetone precipitation)^30^ enabled using the Thermo Kingfisher automated purification system. Following a one-step reduction and alkylation as described above, an equivalent volume of 100% ethanol was added to each sample. Sera-mag beads (equal amounts of hydrophilic and hydrophobic) were washed three times with DI water, and a 250 µg µL^−1^ slurry was prepared in 50% ethanol. Slurry aliquots of 100 µL were distributed to a 96-deep well plate and subsequently washed again for two minutes in 50% ethanol using the Kingfisher automated system. The magnetic beads were then transferred into the sample plate and incubated for 14 min with gentle mixing. The bead-protein mixture was washed three times with 70% ethanol and then transferred to a 350 µL 96 well plate containing 100 µL of 100 mM TEAB pH 8.5 and digested as described above.

### Isobaric labeling

Peptides were labeled with TMT 10-plex reagents for the POC and TMTpro 18-plex for the fractionation experiment according to the manufacturer’s recommendations with slight modifications. Briefly, digests were thawed and tested using pH strips to ensure pH was between 8 to 8.5. The labeling reagents were allowed to equilibrate to room temperature and solubilized with anhydrous acetonitrile for 5 min with occasional vortexing. Each TMT reagent was added to the appropriate sample (Fig. S1) at a ratio of label to the total peptide of 8:1 and a final acetonitrile concentration of 19.5%. Incubation was carried out at room temperature for 1.5 h in the dark and quenched with 0.3% hydroxylamine final concentration for 15 min. The labeled digests were pooled accordingly into four samples (vehicle1, vehicle2, treated1, and treated2) for the POC, and six samples (vehicle1, vehicle2, vehicle3, treated1, treated2 and treated3) for the fractionation set. Each pooled sample was equally split by weight resulting in two duplicate sets of samples. An SDS-solubilized isobarically-labeled Jurkat cell digest (TMT 131 C for the POC and TMT 135 N for the fractionation set) was added to half of the split samples at four times^31,32^ the total protein concentration of the 37 °C sample to act as a trigger channel in the same way as in the BASIL and SCoPE-MS approaches^13–16^. Samples were dried in a SpeedVac and desalted using Pierce peptide desalting spin columns (Thermo Fisher Scientific) according to the manufacturer’s recommendations.

### Offline high pH fractionation

Desalted peptides from the sample set prepared for fractionation were subjected to offline high-pH reversed-phase separation on an Agilent 1290 HPLC using a Phenomenex XBridge BEH C18 Column, 130 Å, 3.5 µm, 3 mm×150 mm (Waters, MA), which was heated to 45 °C. The dried desalted isobarically-labeled digested samples were resuspended in 100 µL of solvent A (20 mM ammonium formate, pH 10, and 5% acetonitrile), and the entire sample was injected onto the column. Separation was accomplished using the following gradient: 3% solvent B (20 mM ammonium formate, 95% acetonitrile, and pH 10) isocratic flow (450 µL min^−1^) for 9 minutes, 3–35% solvent B in 40 min, 35–90% solvent B in 4 minutes, 90% solvent B isocratic flow for 3 min, 90–3% solvent B in 2 min, and 3% solvent B isocratic flow for 20 minutes. The fractions were collected every minute and then concatenated into 24 fractions, dried in a Speedvac, and stored at −80 °C until ready to use.

### nLC MS^2^ analysis

The POC desalted samples were reconstituted in 40 µL of 0.1% TFA, and 2 µL of each sample was injected for each nLC- MS^2^ analysis in technical triplicate. Chromatographic separation was accomplished using an UltiMate 3000 RSLC nLC system equipped with a 2 cm trap column set at 45 °C, and a 50 cm × 75 µm I.D. analytical column set at 45 °C. Samples were loaded for 5 minutes at 3 µL min^−1^ in a 0.1% FA. The reversed-phase gradient started at 1% solvent B (B), which consisted of 0.1% FA in mass spectrometry-grade acetonitrile and held constant for 5 min. Solvent B was increased to 5% in one minute and 23% over the next 90 minutes. A washout period occurred by increasing solvent B to 80% in 3 min and held constant for 2 min. The analytical column was re-equilibrated with 11 µL of solvent A (0.1% FA in MS-grade water). A flow rate of 200 nL min^−1^ was used throughout the entire nLC-MS run.

The fractionated desalted samples were reconstituted in 25 µL of 0.1% FA, and 8 µL of every other fraction (12 total) was loaded into Evosep tips and prepared according to manufacturer’s recommendations. Chromatographic separation was accomplished using an Evosep One equipped with an Evosep 1106 analytical column (Dr. Maisch C18 AQ, 1.9 µm beads, 15 cm × 150 µm ID), and employing the 30 samples per day standard method.

The LC eluent was interfaced to an Orbitrap Exploris 480 mass spectrometer with positive polarity spray voltage set at 2.0 or 2.1 kV for the POC and fractionation sets, respectively. The ion transfer tube was heated to 275 °C. S-lens RF lens % was set at either 45 or 30 for the POC and fractionation set, respectively. Full MS1 scans were acquired using 60 K MS1 resolution setting at mz^−1^ 200, AGC target of 3e^6^, maximum IT of 64 ms, the scan range of 400–1200 mz^−1^, and data collected in profile mode using one microscan. Only one microscan was used for DDA- MS^2^ settings in addition to the AGC target of 1e^6^, isolation width, and offset of 0.7 and 0.3 mz^−1^, respectively. A fixed first mass of 100 mz^−1^ and normalized collision energy of either 34 or 35 for the POC and fractionated sample sets, respectively, was applied to all methods, which collected MS^2^ data in centroid mode. The standard eFT method^33^ allowed a maximum IT of 54 ms, using a resolution setting of 30 K at mz^−1^ 200 and a loop count of 14, while 22 ms was used as the maximum IT at 15 K resolution setting with a loop count of 28 for ΦSDM^9^. The minimum AGC target for data dependent settings in the POC experiment was set to 8e^3^ for all methods. Peptide match set to preferred, exclude isotopes was on, a dynamic exclusion of 30 seconds, and charge exclusion of 1, and > 6 collected in centroid mode. The MS was equipped with a FAIMS Pro interface, and where FAIMS was implemented, three compensation voltages (CVs) were used in standard resolution (65-50-35 for the POC experiment and 70-55-40 for the fractionation sample set). Optimization of FAIMS CVs was conducted initially to determine the highest performing combination of three CVs (Fig. S2)

### Data analysis

Raw files were first analyzed individually using Proteome Discoverer (PD) (version 2.4 for POC and 3.0 for the fractionation set, Thermo Fisher Scientific) with the Sequest HT search engine. Tryptic peptides were included in the searches with a maximum of two missed cleavages. Precursor and fragment mass tolerances were set to 15 ppm and 0.02 Da, respectively. Static modifications included either TMT6plex or TMTpro on the peptide N-terminus and lysine residues and carbamidomethylation of cysteine residues. Oxidation of methionine residues was set as a dynamic modification, along with acetylation of the protein N-terminus. The UniProt human reviewed reference proteome (release 2018_03 or 2022_01 for the POC and fractionation set, respectively) was used. Peptide filtering was set to “high” confidence resulting in a 1% FDR. In the POC experiments, after confirmation that technical replicates (*n* = 3) for each sample within the same iMAATSA had RSD ≤ 15% for protein, peptide, and PSM identifications, triplicates were reprocessed together using the same PD methods for all subsequent analyses.

In the POC experiment, the R-based package TPP^19^ was used for melting curve fitting, and filtering for high-quality melting curves was accomplished, using the following criteria based on the listed experimentally determined parameters: all four samples have a determined melting temperature, slope ≤ -0.06, plateau ≤ -0.3, and R^2^ ≥ 0.8. To further interrogate the data, we developed an R script to analyze data at the protein, PSM, and peptide group levels. Normalization at the protein level was adapted from the existing TPP software. Briefly, the number of proteins selected had to pass the initial quality criteria stated above. Next, we selected proteins present in all iMAATSA and chose the experiment with the greatest number of melting curves. Afterward, median fold changes were calculated for each reporter ion channel, and these were fit using a sigmoidal fit. Finally, the curve with the greatest R^2^ value was used as a correction factor to apply to all the data over the entire temperature range. The MS proteomics data have been deposited to the ProteomeXchange Consortium via the PRIDE^34^ partner repository with the dataset identifiers PXD034087 and PXD040284 for the POC and fractionation experiments, respectively. Additional experimental details about materials, reagents, and methods, including cell culture, sample processing, LC conditions, mass spectrometry parameters, and data analysis, are provided in the Supporting Information file.

In the fractionation experiment, an in-house virtual basic (VBA) script was used to process the triplicate data sets. Each replicate treatment (“DMSO” or “TREATED”) was normalized to the 37 °C channel. Corrected ratios were imported into Spotfire (TIBCO, version 11.4), and the T_m_ for each replicate treatment was determined using the “logistic regression curve fit” function. Melting-curves were assessed and filtered out by removing proteins, which had a T_m_ of 37 °C or below and that failed to have a calculated T_m_ for at least two out of the three melting-curves for both treatments.

## Supplementary information

Supplemental Information

Description of Additional Supplementary Files

Supplementary Data file 1

Supplementary Data file 2

Supplementary Data file 3

Supplementary Data file 4

Supplementary Data file 5

Supplementary Data file 6

Supplementary Data file 7

Supplementary Data file 8

Supplementary Data file 9

Supplementary Data file 10

Supplementary Data file 11

Supplementary Data file 12

Supplementary Data file 13

Supplementary Data file 14

## Data Availability

The raw mass spectrometry data files and Proteome Discoverer results that were generated and analyzed in the current study are available through the ProteomeXchange Consortium via the PRIDE partner repository with the dataset identifiers PXD034087 and PXD040284. All other data including supporting figures and Supplementary Data files 1-14 are available within the paper.
