# Supplementary material for: Experimental strategies to improve drug-target identification in mass spectrometry-based thermal stability assays
Source: Commun Chem. 2023 Apr 6;6:64. doi: 10.1038/s42004-023-00861-1 (PMC10079678; doi:10.1038/s42004-023-00861-1)
Supplement: Supplementary file 1 — Supplemental Information [file 42004_2023_861_MOESM1_ESM.pdf]

Supplementary Information

Title

Experimental strategies to improve drug-target identification in mass spectrometry-based thermal stability assays

Clifford G. Phaneuf[1,2], Konstantin Aizikov[3], Dmitry Grinfeld[3], Arne Kreutzmann[3], Daniel Mourad[3], Oliver Lange[3], Daniel Dai[2], Bailin Zhang[2], Alexei Belenky[4], Alexander A. Makarov[3], and Alexander R. Ivanov[1]*

1 Barnett Institute of Chemical and Biological Analysis, Department of Chemistry and Chemical Biology, Northeastern University, 360 Huntington Ave., Boston, Massachusetts 02115, United States

2 Sanofi, Translational Sciences, 640 Memorial Drive, Cambridge, Massachusetts

3 Thermo Fisher Scientific, Hanna-Kunath-Str. 11, Bremen, 28199, Germany

4 Tarmeta Biosciences, 27 Strathmore Rd, Natick, Massachusetts 01760

*Corresponding author: *Dr. Alexander R. Ivanov,* email: *a.ivanov@northeastern.edu*, Tel: 1-617-373-6549

Supplementary Figure 1

a

℃ HEATED	DMSO TMT channel	MEKi TMT channel
37	130N	134N
41	129C	133C
46	129N	133N
50	128C	132C
55	128N	132N
59	127C	131C
64	127N	131N
68	126	130C
SIILCC	135N	

b

	℃ HEATED	TMT channel
DMSO-1 DMSO-2 MEKi-1 MEKi-2	37	126
	41	127N
	44	127C
	47	128N
	50	128C
	53	129N
	56	129C
	59	130N
	63	130C
	67	131N
SIILCC		131C

Figure S1. Heat treatment and isobaric labelling schemes for the proof-of-concept (a) and fractionation (b) sample sets.

The iMAATSAs, either applying or not applying the FAIMS Pro interface, are labeled as "F" or "nF," respectively.

Supplementary Figure 2

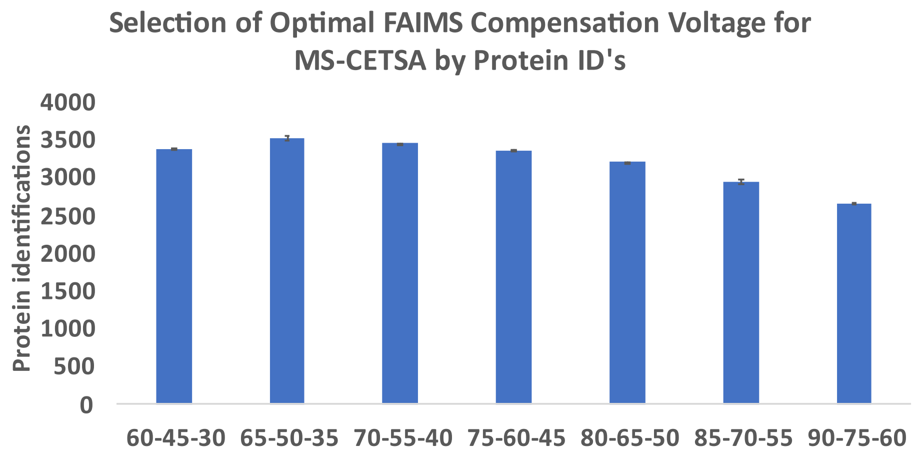

Figure S2. FAIMS compensation voltages (CV) Optimization. Protein identifications (y-axis) vs. combinations of three CV settings (x-axis).

Supplementary Figure 3

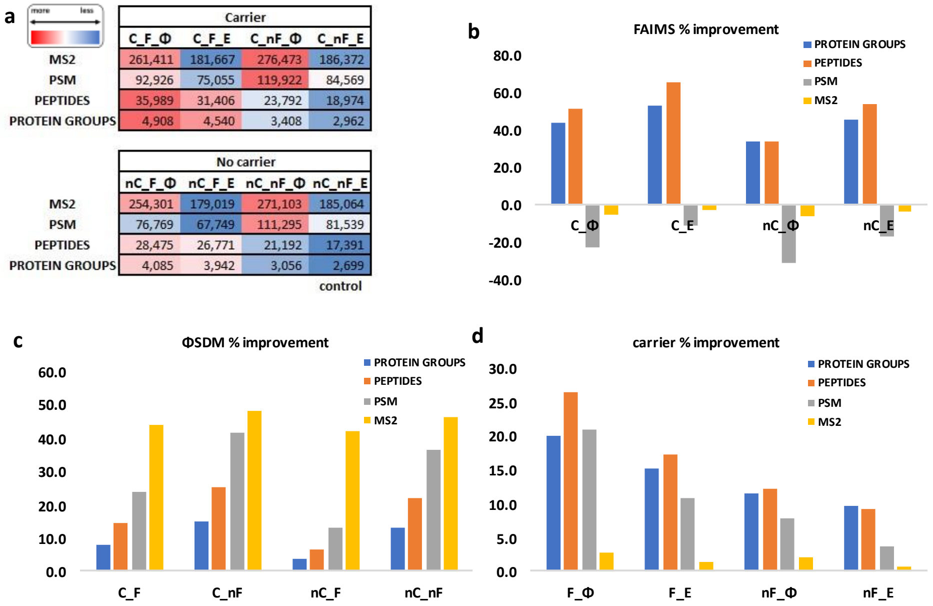

Figure S3. (a) Heatmap of proteomic identifications for all iMAATSA. Row-wise comparison are color-coded. The C_F_Φ column was shown to have the most collective proteomic identifications. Proteomic identifications percent improvement evaluation for (b) ΦSDM, (c) FAIMS, and (d) SIILCC. Percent improvement was calculated for ΦSDM, FAIMS and SIILCC individually for protein groups, peptides, PSM, and MS2 identification. An example of the ΦSDM percent improvement calculation of protein groups for C_F is the following: [[C_F_Φ (261,411) – C_F_E (181,667)] / C_F_E (181,667)] x 100.

Supplementary Figure 4

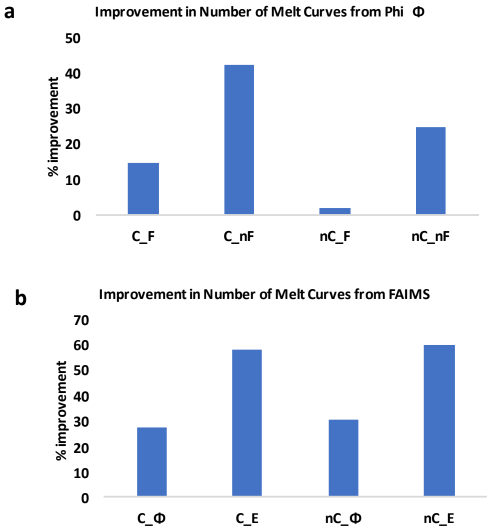

Figure S4. High-quality melting curves percent improvement evaluation of (a) ΦSDM and (b) FAIMS. Percent improvement is calculated similarly as in Supplementary Figure 1. High-quality protein melting-curves have a R^2 "goodness-of-fit" statistic value of ≥ 0.8, the slope of the inflection point ≤ -0.06, a lower plateau value of ≤ 0.3, and the treated and control melting temperatures must be present for both biological replicates.
