# Supplementary material for: Experimental strategies to improve drug-target identification in mass spectrometry-based thermal stability assays
Source: Commun Chem. 2023 Apr 6;6:64. doi: 10.1038/s42004-023-00861-1 (PMC10079678; doi:10.1038/s42004-023-00861-1)
Supplement: Supplementary file 2 — Description of Additional Supplementary Files [file 42004_2023_861_MOESM2_ESM.pdf]

Description of Additional Supplementary Files

File name: Supplementary Data 1

Description: (C_F_Φ) Protein identification and quantitative data from the POC experiment where a carrier channel, FAIMS, and ΦSDM was implemented (including duplicate treated and control experiments)

File name: Supplementary Data 2

Description: (C_F_E) Protein identification and quantitative data from the POC experiment where a carrier channel, FAIMS, and eFT was implemented (including duplicate treated and control experiments)

File name: Supplementary Data 3

Description: (C_nF_Φ) Protein identification and quantitative data from the POC experiment where a carrier channel, no FAIMS, and ΦSDM was implemented (including duplicate treated and control experiments)

File name: Supplementary Data 4

Description: (C_nF_E) Protein identification and quantitative data from the POC experiment where a carrier channel, no FAIMS, and eFT was implemented (including duplicate treated and control experiments)

File name: Supplementary Data 5

Description: (nC_F_Φ) Protein identification and quantitative data from the POC experiment where no carrier channel, FAIMS, and ΦSDM was implemented (including duplicate treated and control experiments)

File name: Supplementary Data 6

Description: (nC_F_E) Protein identification and quantitative data from the POC experiment where no carrier channel, FAIMS, and eFT was implemented (including duplicate treated and control experiments)

File name: Supplementary Data 7

Description: (nC_nF_Φ) Protein identification and quantitative data from the POC experiment where no carrier channel, no FAIMS, and ΦSDM was implemented (including duplicate treated and control experiments)

File name: Supplementary Data 8

Description: (nC_nF_E) Protein identification and quantitative data from the POC experiment where no carrier channel, no FAIMS, and eFT was implemented (including duplicate treated and control experiments)

File name: Supplementary Data 9

Description: (C_F_Φ) Protein identification and quantitative data from the fractionation experiment where a carrier channel, FAIMS, and ΦSDM was implemented (treated and control replicate 1)

File name: Supplementary Data 10

Description: (C_F_Φ) Protein identification and quantitative data from the fractionation experiment where a carrier channel, FAIMS, and ΦSDM was implemented (treated and control replicate 2)

File name: Supplementary Data 11

Description: (C_F_Φ) Protein identification and quantitative data from the fractionation experiment where a carrier channel, FAIMS, and ΦSDM was implemented (treated and control replicate 3)

File name: Supplementary Data 12

Description: (nC_nF_E) Protein identification and quantitative data from the fractionation experiment where no carrier channel, no FAIMS, and eFT was implemented (treated and control replicate 1)

File name: Supplementary Data 13

Description: (nC_nF_E) Protein identification and quantitative data from the fractionation experiment where no carrier channel, no FAIMS, and eFT was implemented (treated and control replicate 2)

File name: Supplementary Data 14

Description: (nC_nF_E) Protein identification and quantitative data from the fractionation experiment where no carrier channel, no FAIMS, and eFT was implemented (treated and control replicate 3)
